# Report of Committee on Practical Medicine

**Published:** 1859-01

**Authors:** F. K. Bailey

**Affiliations:** Chairman


[Continued from page 660.]

"I do not wish to make a hobby, or laud any remedy more than it requires, but must say that I am convinced that veratrum is one of our great therapeutical remedies; and as such, demands our attention and administration in most inflammatory diseases.

"In reply to question No. 2, I would say, that scarlatina has prevailed nearly all the year, and is still prevailing at this time within six or eight miles of our place. During the summer and fall, and early part of winter, nothing unusual characterized its type; in fact, if I should regard anything unusual about the disease, I should say it was unusually mild.

"This mildness of type continued until January. No deaths had occurred, although I had cases all the time. The first cases in our vicinity that proved malignant and fatal, occurred in Toulon, a village six miles distant. The patients (two children), under the care of a homœopath, were sick only a few days, and, as I learned, died with symptoms resembling those in arachnitis.

"On the 17th of same month I was called to see a boy, aged eleven years. The rash was fully out, but looked purple; he seemed a little incoherent; pulse quick and weak. I ordered lukewarm spongings to the surface, cold applications to the head, warm pediluvia, to be succeeded by sinapisms to the feet, a mild dose of oleum ricini, and when it operated to be followed by spts. nit. dulc. ℥ij., comp. syr. scillæ ℥j., mix, and give ʒj. every two hours. On the next morning I saw him again; symptoms but little changed. Oil had operated. Treatment not changed. P. M., saw him again; symptoms materially changed for the worse; constant muttering delirium; sunken appearance of the countenance; cannot recognize friends; floccitatio; evacuations passing in the bed without his knowledge. Ordered quinine, with carb. ammonia, every two hours; and to subdue the pulse, ordered tr. veratrum, in diminished doses, every three hours. Spts. nit. and tr. scillæ comp. to be suspended.

"Next morning much worse; treatment continued, but he died the succeeding night.

"While at this house, my attention was called to an infant, aged one year, who had only been sick an hour or two. I made a prescription, but ere fourteen hours had passed they both lay corpses in the same house.

"This child sunk as if by some sedative poison. In two other houses in the same neighborhood seven other cases occurred, and out of that number only four recovered. Some died with disease of the glands of the throat and ulceration of the tonsils, some with an erysipelatous affection of the eyes and face, but most cases died as if from meningitis."

The Doctor continues by saying that on the 6th February scarlatina entered his family. Five children of his own, and a girl of thirteen residing in the family, were sick, and one died. Those who recovered were sick a long time, and the convalescence protracted.

In the case of his son, aged ten, the thumb nails, some of the finger nails, and the entire surface of the skin sloughed off.

After acknowledging his obligations to Drs. Secord and Clark of Galva, and Drs. Fitch and Cleveland of Kewanee, for medical aid during the sickness of his children, the Doctor adds:

"I am under the painful necessity of acknowledging that I have but little confidence in medication in this malignant disease. I have tried the mild and expectant plan, I have tried the stimulant plan, the cold and warm and the sedative plans, tr. veratrum, with the different liniments and poultices to the throat, with the multiplicities of gargles to the tonsils, without being able to decide upon the superior virtues of any one course."

In reply to question No. 3, the Doctor writes:

"Typhoid fever, during the year 1857, has not prevailed as much as usual. What cases I have seen were unusually tedious. No cases have proved fatal under my care. I see no well-marked distinctions between cases occurring in the timber or on the prairie. This is a rich prairie region, the timber being on the low ground and the prairie high and rolling.

"Milk-sickness is not known in this vicinity; and as for answering to its origin I shall not attempt it, as it has baffled our best pathologists and chemists up to the present time.

"Cholera infantum prevails every summer in this region. I believe it is produced by teething and the excessive warm weather, unwholesome atmosphere, sleeping in crowded and illy ventilated bedrooms, and not by miasmatic causes; and consequently think that anti-periodic remedies are of little use in any stage of the disease. For a topographical description of this section of Illinois, see a letter from the undersigned, published in the *Transactions* for the year 1856."

The report of your committee is hereby respectfully presented, and with the hope that the several subjects treated will receive further attention from future committees.

F. K. BAILEY, *Chairman.*

